# The Relationship Between Immunogenic Cell Death and Cellular Senescence in Cancer Immunotherapy

**DOI:** 10.3390/life16091536

**Published:** 2026-09-15

**Authors:** Seong-Ah Shin, Minji Kim, Moonsu Kim, Seyeon Choi, Hyun Ho Park, Chang Sup Lee

**Affiliations:** 1 College of Pharmacy and Research Institute of Pharmaceutical Sciences, Gyeongsang National University, Jinju 52828, Republic of Korea; shinsaya@gnu.ac.kr (S.-A.S.); minji8038@gnu.ac.kr (M.K.); kk10022@gnu.ac.kr (M.K.); seyeon0608@gnu.ac.kr (S.C.); 2 College of Pharmacy, Chung Ang University, Seoul 06974, Republic of Korea; xrayleox@cau.ac.kr

**Keywords:** immunogenic cell death, cellular senescence, cancer immunotherapy, immune checkpoint inhibitors, tumor microenvironment, senescence-associated secretory phenotype, cGAS–STING signaling, antitumor immunity

## Abstract

Despite rapid advances in anticancer therapy, cancer incidence and mortality remain substantial worldwide. Although immune checkpoint inhibitors (ICIs), including anti-PD-1/PD-L1 and anti-CTLA-4 therapies, have transformed cancer treatment by harnessing host antitumor immunity, their efficacy remains limited to a subset of patients, largely because of the complexity of the tumor microenvironment. This review examines drug-induced immunogenic cell death (ICD) and drug-induced senescence as complementary strategies for overcoming these limitations and enhancing immunotherapeutic responses. We further highlight evidence that agents capable of inducing ICD may instead promote cellular senescence when administered at lower concentrations for prolonged periods, indicating that these distinct cellular outcomes can be determined by drug dose and treatment duration. This dose- and time-dependent relationship suggests that the therapeutic application of the same agent may require optimization according to the patient’s condition and tumor stage. Collectively, this review provides an integrated perspective on the selective use of ICD and senescence induction to enhance antitumor immunity, improve therapeutic outcomes, and potentiate synergistic responses to existing ICIs.

## 1. Introduction

Cancer remains a leading cause of mortality worldwide and an unresolved public health challenge [1]. In 2022, approximately 20 million new cancer cases and 9.7 million cancer-related deaths were reported globally [2]. The number of new cases is projected to reach approximately 35 million by 2050, driven by factors including population aging, smoking, obesity, and physical inactivity, which are altering the prevalence of established cancer risk factors [2]. Despite substantial advances in cancer therapy, the persistently high incidence and mortality rates underscore the continued need to develop more effective treatment strategies [3].

In recent years, cancer treatment has progressed considerably beyond traditional cytotoxic agents through the development of targeted therapies and immunotherapies [4]. Unlike conventional chemotherapy, which nonspecifically targets both tumor and normal proliferating cells and thereby causes toxic effects, and targeted therapies, which inhibit specific oncogenic pathways within tumor cells and act directly on tumor cells, cancer immunotherapies harness the patient’s immune system to generate antitumor responses within the tumor microenvironment (TME) [5]. Immunotherapies are also generally associated with lower toxicity and can provide prolonged antitumor efficacy in the clinical setting [6]. Nevertheless, despite the transformative impact of cancer immunotherapies, including immune checkpoint inhibitors (ICIs), only a subset of patients derives meaningful benefit because of the complexity and heterogeneity of the TME [7].

Based on the extent of CD8^+^ T-cell infiltration and inflammation within the TME, tumors are broadly classified as hot (T-cell-infiltrated and inflamed) or cold (T-cell non-infiltrated and non-inflamed) [7]. Hot tumors, characterized by abundant CD8^+^ T-cell infiltration, generally respond more effectively to ICIs targeting PD-1/PD-L1 and CTLA-4 [8], whereas cold tumors lacking CD8^+^ T-cell infiltration are immunologically quiescent and less responsive to immunotherapy [9]. Accordingly, considerable research has focused on converting cold tumors into hot tumors by reprogramming the TME or promoting immune cell activation to enhance immunotherapeutic efficacy [10]. Indeed, certain anti-cancer chemotherapeutic agents could contribute to this cold-to-hot transition by inducing tumor cell responses that enhance tumor-associated inflammation and immune activation [11,12]. In this process, stressed tumor cells facilitate the recruitment and activation of antigen-presenting cells (APCs), particularly dendritic cells (DCs), by exposing or releasing tumor-derived antigens and immunostimulatory signals [13]. Activated APCs then promote antigen presentation and the priming of tumor-specific CD8^+^ T cells [14]. This leads to increased infiltration and activation of effector CD8^+^ T cells, strengthening antitumor immunity and rendering the tumor more responsive to immunotherapy [15]. In this context, drug-induced immunogenic cell death (ICD) and drug-induced senescence represent two important approaches capable of stimulating antitumor immunity through T-cell activation. Notably, recent studies indicate that certain chemotherapeutic agents, including doxorubicin, can induce distinct cellular responses depending on dose and treatment duration: lower doses tend to promote senescence in human cancer cells, whereas higher doses induce ICD [16,17]. Therefore, this review delineates the mechanisms underlying drug-induced ICD and senescence, examines their relationship, and discusses strategies for exploiting these processes to optimize combination immunotherapy.

## 2. ICD and Immunotherapy

ICD is a form of cell death that elicits coordinated innate and adaptive immune responses following treatment with specific chemotherapeutic agents, including doxorubicin, mitoxantrone, and 5-fluorouracil, as well as photodynamic therapy and radiotherapy [18,19,20,21,22]. Recent studies suggest that ICD may enhance the efficacy of cancer immunotherapy, particularly because many solid tumors exhibit an immunologically ‘cold’ phenotype characterized by limited cytotoxic T-cell infiltration within the TME [9,23].

### 2.1. Representative Hallmarks of ICD

**Figure 1:**
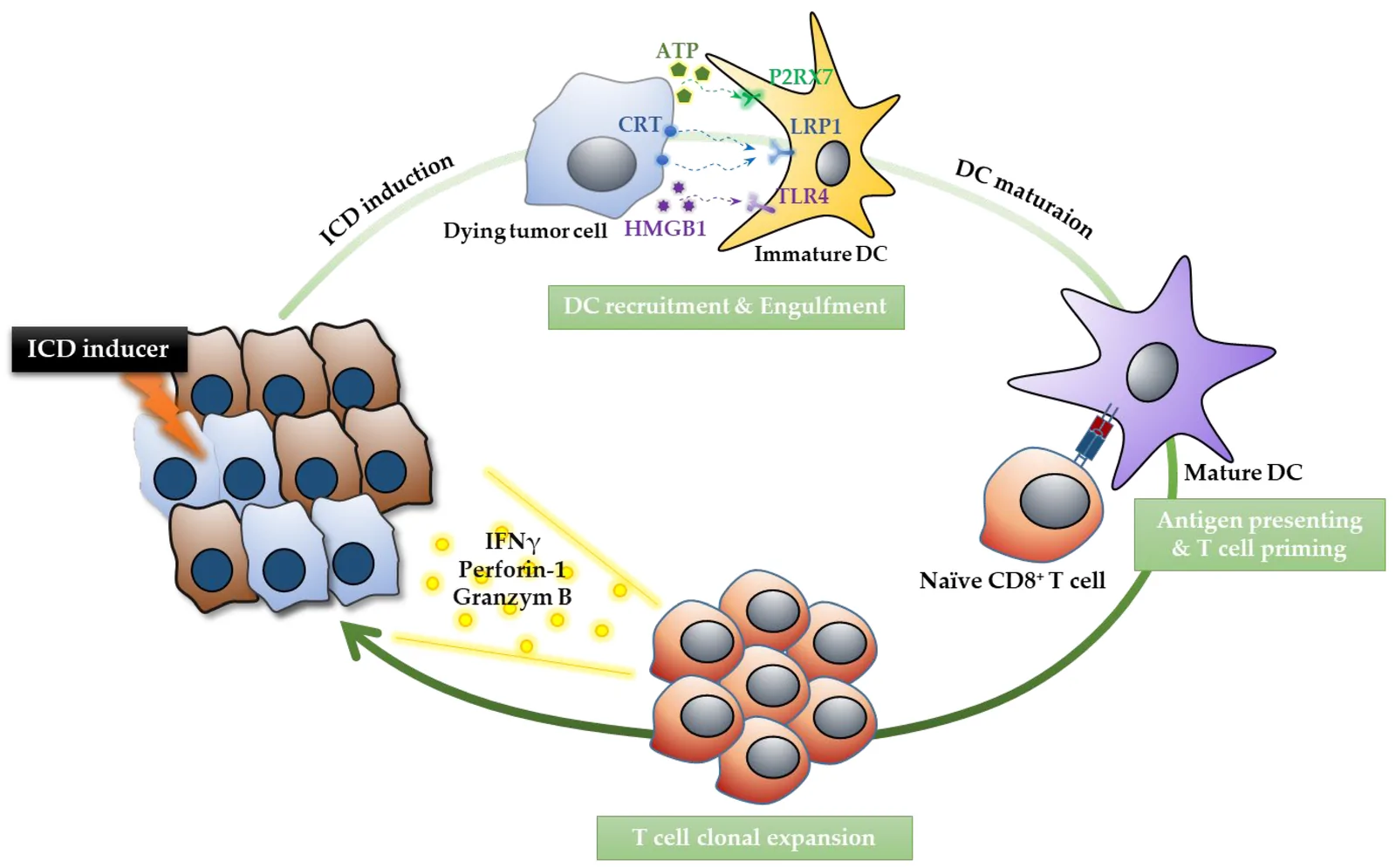
Characteristics of immunogenic cell death (ICD). Cancer cells undergoing ICD expose calreticulin (CRT) on the cell surface as an ‘eat-me’ signal that promotes their engulfment by dendritic cells (DCs) via low-density lipoprotein receptor-related protein 1 (LRP1), and release ATP and high mobility group box 1 (HMGB1) as damage-associated molecular patterns (DAMPs). ATP drives DC recruitment and activation through purinergic receptor (P2RX7) signaling, whereas HMGB1 facilitates DC maturation through Toll-like receptor 4 (TLR4). Collectively, these events promote cytotoxic T-cell priming, thereby eliciting a robust tumor-specific immune response.

Cancer cells undergoing chemotherapy-induced ICD expose calreticulin (CRT) on the cell surface and release damage-associated molecular patterns (DAMPs) as intracellular danger signals, promoting the recruitment and maturation of APCs, particularly dendritic cells (DCs) (Figure 1) [24,25]. Mature DCs subsequently activate cytotoxic T lymphocytes (CD8^+^ T cells), thereby promoting tumor-specific innate and adaptive immune responses [25].

CRT is a well-established chaperone protein that primarily resides in the endoplasmic reticulum (ER) but can serve as an immunogenic ‘eat-me’ signal when exposed on the cell surface [26,27,28]. Defective CRT exposure prevents the engulfment of dying cancer cells by DCs and can impair their immunogenicity in mice [29]. Thus, CRT is considered a critical determinant of immune responses induced by immunogenic chemotherapy.

ICD-associated DAMPs also include ATP and High Mobility Group Box 1 (HMGB1), which are released in response to intracellular stress signaling [13]. Extracellular ATP functions as a ‘find-me’ signal by binding P2RX7 receptors on DCs and activating the NLRP3 inflammasome, thereby inducing IL-1β production, which is essential for the activation of IFNγ-producing T cells [30,31,32]. HMGB1 is a nonhistone chromatin protein that normally resides in the nucleus, where it interacts with DNA and regulates transcription. Following cellular damage, HMGB1 is released and binds multiple pattern-recognition receptors (PRRs), including the advanced glycation end-product-specific receptor (AGER, also known as RAGE) and Toll-like receptor 4 (TLR4) [13,33]. TLR4 signaling has been reported to be sufficient for ICD [34], whereas HMGB1 knockout in tumors or TLR4 blockade in vivo reduces the therapeutic efficacy of ICD inducers [35].

CRT exposure and DAMP release are among the most widely used molecular hallmarks for identifying potential ICD inducers in vitro. However, the presence of these markers alone is not sufficient to establish that functional ICD has been induced. Functional ICD is more convincingly supported when these molecular hallmarks are accompanied by downstream immune activation, including the engulfment of dying tumor cells by DCs, DC maturation and antigen presentation, and the subsequent activation of tumor-specific T cells [36,37]. Accordingly, molecular hallmarks should be regarded as indicators of immunogenic cellular stress rather than as definitive evidence of functional immunogenicity, and these limitations should therefore be taken into account when interpreting the agents summarized in this review.

### 2.2. Signaling Pathway of ICD

ICD inducers generally impose ER stress on target cells, and accumulating evidence links ER stress to ICD-associated CRT exposure [25,38,39]. Under ER stress, unfolded proteins sequester the luminal ER chaperone GRP78/Bip, activating three ER transmembrane stress sensors: inositol-requiring enzyme 1α (IRE1α), protein kinase RNA-like ER kinase (PERK), and activating transcription factor 6 (ATF6) [40]. Among these sensors, PERK plays a critical role in ICD-associated CRT exposure [41].

**Figure 2:**
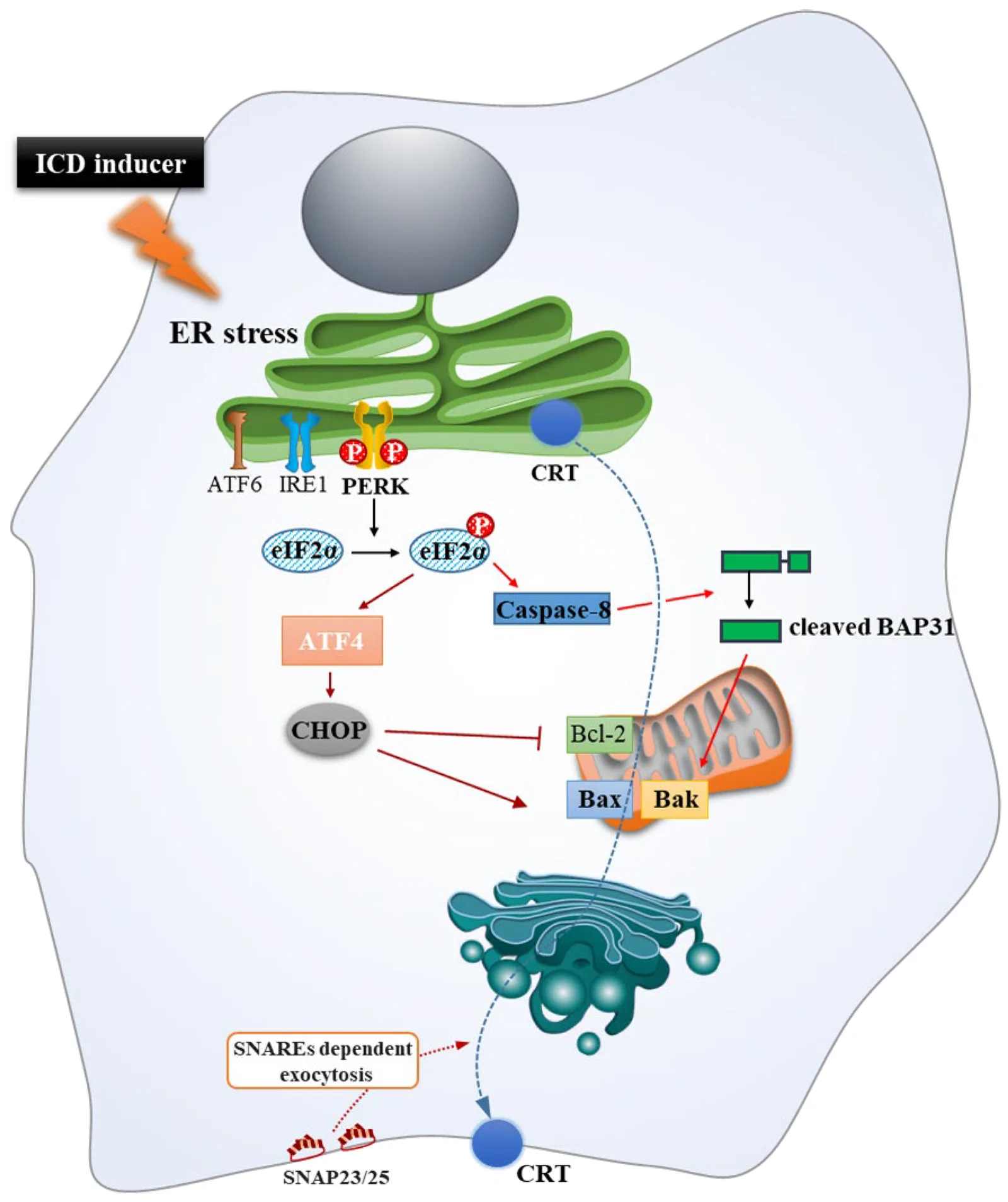
Signaling pathways underlying CRT exposure in dying cancer cells induced by ICD inducers. Exposure to ICD-inducing stress disrupts endoplasmic reticulum (ER) homeostasis and activates the unfolded protein response (UPR). This process activates protein kinase R-like endoplasmic reticulum kinase (PERK), which phosphorylates the eukaryotic translation initiation factor 2α (eIF2α) and initiates downstream ER stress signaling. Subsequent events include caspase 8-mediated cleavage of B-cell receptor-associated protein 31 (BAP31) and activation of the pro-apoptotic proteins BCL2-associated X protein (Bax) and Bcl-2 homologous antagonist/killer (Bak). CRT subsequently traffics to the Golgi apparatus and is transported to the cell surface through a SNARE-dependent exocytic pathway.

As shown in Figure 2, upon ER stress, PERK oligomerizes and undergoes trans-autophosphorylation, which stabilizes the active conformation of its cytosolic kinase domain. Activated PERK then phosphorylates its principal substrate, eukaryotic translation initiation factor 2α (eIF2α) [42]. Phosphorylated eIF2α subsequently promotes translation of activating transcription factor (ATF4), leading to induction of C/enhancer binding protein-homologous protein (CHOP), a key pro-apoptotic factor [42,43]. CHOP downregulates the anti-apoptotic protein Bcl-2 while upregulating the pro-apoptotic proteins Bim, Puma, and Bax, ultimately promoting caspase-3 cleavage and apoptosis [44,45]. In parallel, eIF2α phosphorylation is followed by partial caspase-8 activation and cleavage of the ER protein B-cell receptor-associated protein 31 (Bap31), which subsequently activates BCL2-associated X protein (Bax) and Bcl-2 homologous antagonist/killer (Bak) [41]. CRT translocation to the cell surface requires anterograde ER-to-Golgi trafficking followed by SNARE-dependent exocytosis involving vesicle-associated SNAREs, such as VAMP1, and plasma membrane SNAREs, including SNAP23/25 [41]. Although PERK-mediated ER stress signaling is required for CRT exposure, the precise molecular mechanism linking PERK activation to CRT translocation remains incompletely understood. During ICD, surface-exposed CRT functions as an “eat-me” signal that facilitates phagocytosis of dying cancer cells by DCs [46]. Concurrently, released DAMPs promote DC activation and upregulation of maturation markers, including CD80, CD86, and major histocompatibility complex class (MHC) II [47]. Activated DCs secrete pro-inflammatory cytokines, including IL-1β and TNF-α, and prime tumor-specific CD8^+^ T lymphocytes, ultimately promoting IFNγ production and cancer cell elimination [47]. Thus, ICD induction can synergize with ICIs, including anti-PD-1/PD-L1 and anti-CTLA-4 therapies, to strengthen antitumor immune responses and enhance immunotherapeutic efficacy.

### 2.3. Development of ICD Inducers for Cancer Immunotherapy

The development of ICD inducers represents an attractive strategy for converting immunologically ‘cold’ tumors into ‘hot’ tumors and thereby enhancing synergy with cancer immunotherapy. However, many chemotherapeutic agents known to induce ICD, including doxorubicin, mitoxantrone, and oxaliplatin, are first-generation anticancer drugs associated with substantial toxicity and adverse effects because they lack selectivity for cancer cells over normal cells [4,48]. To improve the safety and efficacy of ICD induction, numerous studies have explored innovative approaches, including structural modification to reduce the required drug dose and nanotechnology-based strategies for targeted delivery to tumor sites [49]. Despite advances in the development of ICD inducers, most remain at an early stage of investigation and are not yet suitable for routine clinical application, leaving several challenges unresolved. Nevertheless, drug-induced ICD remains a promising strategy for improving cancer treatment.

## 3. Drug-Induced Senescence and Immune Response

**Figure 3:**
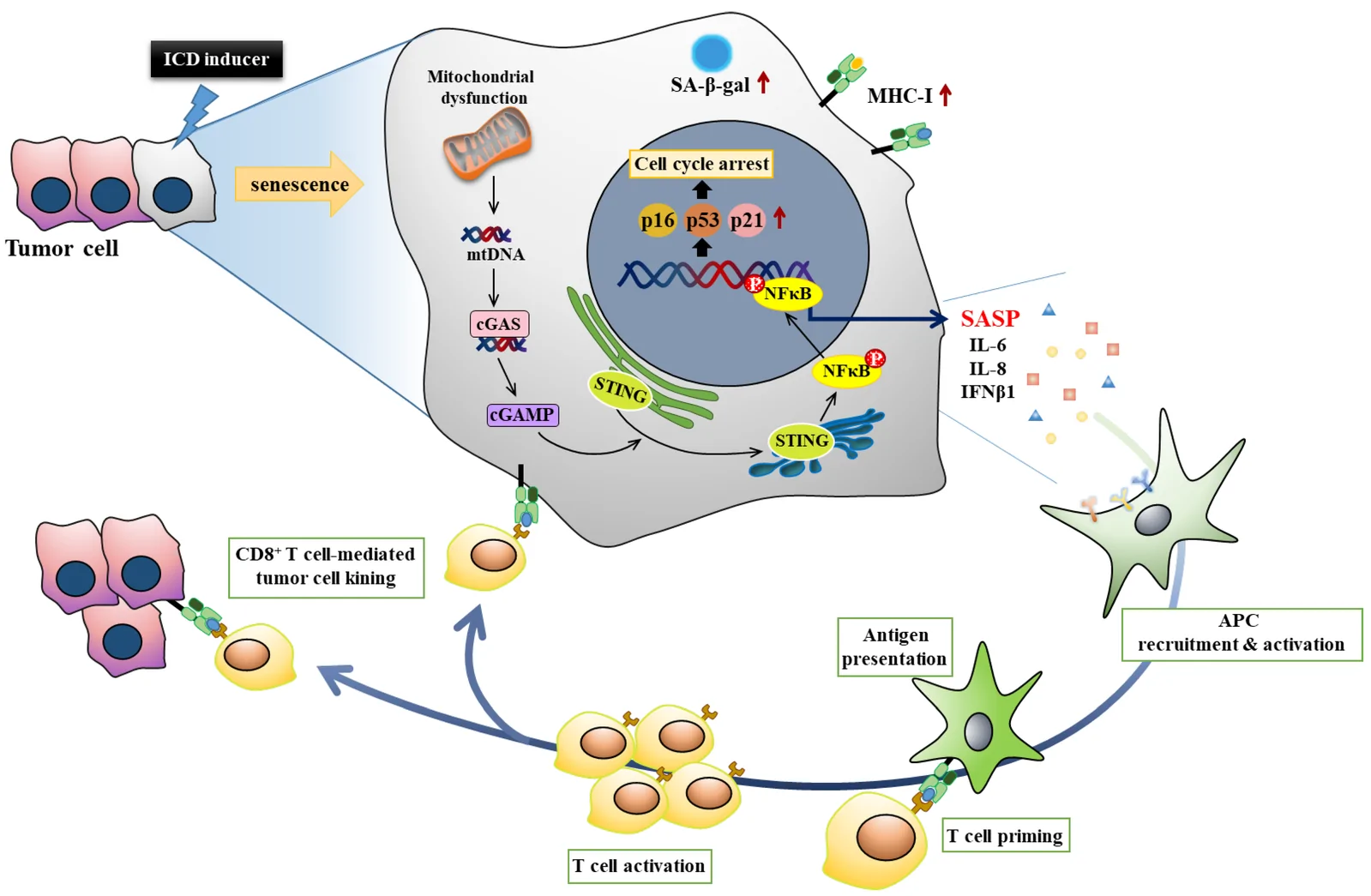
Mechanism of drug-induced senescence. Senescent cells induced by ICD inducers upregulate major histocompatibility complex class I (MHC-I) and related antigens. Through the senescence-associated secretory phenotype (SASP), regulated by nuclear factor-κB (NF-κB) and the cyclic GMP-AMP synthase (cGAS)-STING pathway, senescent cells recruit and promote the maturation of antigen-presenting cells (APCs), including DCs. Activated APCs subsequently promote T-cell priming and antitumor immune responses.

Drug-induced senescence (Figure 3) is a form of therapy-induced premature aging in cancer cells and represents an emerging anticancer strategy with potentially lower nonspecific toxicity than conventional cytotoxic agents, including ICD inducers [50]. Senescence induction has been extensively studied with alkylating agents, topoisomerase inhibitors, and microtubule inhibitors [51]. Cellular senescence is characterized by stable, irreversible cell-cycle arrest that prevents the proliferation of damaged or stressed cells while preserving metabolic activity [52]. This state is primarily mediated by the p53/p21WAF1/CIP1 and p16INK4A/pRB tumor suppressor pathways [52]. DNA damage or oncogenic stress activates p53, inducing p21 and suppressing cyclin-dependent kinase (CDK) activity, whereas p16 reinforces Rb-mediated G1 arrest through inhibition of CDK4/6 [53,54]. Unlike quiescence, senescence persists after removal of stimuli such as growth factors or mitogenic signals, a property that may help prevent cancer cell recurrence [55]. Certain anticancer agents, including ICD inducers, can also induce senescence when administered at low concentrations for prolonged periods. Senescent cancer cells not only undergo irreversible cell-cycle arrest but also influence neighboring tumor cells and recruit immune cells through the senescence-associated secretory phenotype (SASP) [56]. This senescence-associated state can contribute to antitumor activity by restricting tumor growth and enhancing immune surveillance [57]. Moreover, senescent cancer cells can upregulate specific immune ligands that promote the recruitment and activation of immune populations, including CD4^+^ T cells, CD8^+^ T cells, and Th17 cells, thereby facilitating antitumor immune responses [58]. Thus, drug-induced senescence may exert anticancer effects through the combined induction of durable tumor cell-cycle arrest and activation of multiple components of the immune system.

### 3.1. Mechanism of Drug-Induced Senescence: SASP and the Cyclic GMP-AMP Synthase (cGAS)-STING Signaling Pathway

Senescent cells exhibit a pro-inflammatory phenotype characterized by the SASP, which comprises pro-inflammatory cytokines and chemokines (IL-1α, IL-6, IL-1β, IL-8, CXCL1, CXCL2, and CCL2), growth factors (amphiregulin, FGF, and TGF-β), and extracellular matrix-remodeling factors (MMP1, PAI-1, and TIMP-2). These factors can induce senescence in surrounding cells and recruit immune cells [59,60,61,62]. The SASP may therefore promote immune-cell accumulation within the TME and enhance cytotoxic responses against therapy-induced senescent tumor cells [63]. SASP production is driven, in part, by nuclear factor-κB (NF-κB) activation through the cGAS-STING signaling pathway [56].

The cGAS-STING pathway plays a central role in promoting inflammation in senescent cells [64]. During cellular senescence, damaged double-stranded DNA (dsDNA) accumulates in the cytoplasm and is recognized by cGAS as a DAMP [65]. Activated cGAS synthesizes cyclic GMP-AMP (cGAMP), which binds STING and induces its conformational activation and translocation from the ER to the Golgi apparatus [66]. STING subsequently recruits TANK-binding kinase 1 (TBK1) and IκB kinase (IKK), which activate IFN regulatory factor 3 (IRF3) and NF-κB, respectively, thereby promoting the production of type I interferons and inflammatory cytokines [67].

### 3.2. SASP-Mediated Synergy Between Drug-Induced Senescence and Immunotherapy in Tumor Cells

Cellular senescence frequently arises during cancer treatment, and persistent senescent tumor cells can influence tumor progression through the secretion of SASP factors [68]. The SASP recruits CD8^+^ T cells and natural killer (NK) cells and modulates macrophage polarization, thereby promoting the elimination of senescent tumor cells and suppressing tumorigenesis [69]. However, the SASP produced by senescent tumor cells can also modulate therapeutic responses in both cancer and normal cells, either suppressing or promoting tumor formation and normal tissue damage [70]. Furthermore, senescent cells that persist owing to inefficient immune clearance may exert detrimental effects [71]. Their accumulation, together with the continued secretion of SASP factors, can establish a chronic inflammatory microenvironment that promotes tumor cell proliferation, invasion, angiogenesis, and post-therapeutic recurrence [71,72,73]. The balance between the tumor-suppressive and tumor-promoting effects of drug-induced senescence is therefore not fixed, but is shaped by the composition and duration of the SASP as well as by the efficiency with which senescent cells are cleared by the immune system [74,75]. Therapeutic exploitation of the SASP may thus represent a promising strategy for cancer immunotherapy, provided that the duration of the senescent state is controlled and that its potential tumor-promoting effects are taken into account.

## 4. Drug-Induced ICD and Senescence in the TME

Immunotherapy has emerged as a transformative approach to cancer treatment; however, its efficacy is largely restricted to patients with immunologically “hot” tumors characterized by abundant cytotoxic T-cell infiltration within the heterogeneous TME. In contrast, many cancers exhibit a “cold,” non-immunogenic phenotype and consequently respond poorly to immunotherapy. Accordingly, considerable research has focused on converting cold tumors into hot tumors by modulating the TME.

Recent studies have identified ICD induction using established first-generation anticancer drugs as one strategy for promoting this transition. However, many ICD inducers, including doxorubicin, are also toxic to normal cells, highlighting the need to overcome their limited therapeutic selectivity. Notably, the same anticancer agent may induce cytotoxic ICD at high concentrations and short exposure times, whereas lower concentrations and prolonged treatment can induce drug-induced senescence with reduced toxicity while still stimulating immune responses [76]. Therefore, we examined whether various ICD-inducing agents can also promote drug-induced senescence and SASP-mediated immune activation when administered at lower concentrations.

### 4.1. Particular Chemotherapeutics Trigger ICD and Senescence Depending on Concentration and Treatment Duration

Doxorubicin, an anthracycline, is widely used to treat breast, ovarian, gastric, and lung cancers [77,78,79]. Its cytotoxic effects involve apoptosis, calcium dysregulation, oxidative stress, and mitochondrial dysfunction [80]. In CT26 colon cancer cells, doxorubicin treatment at 25 µM for 24 h induces ICD and increases CD4^+^ and CD8^+^ T-cell infiltration [81,82,83]. In contrast, treatment of HCT116 colon cancer cells with a lower concentration of 0.1 µM for 4–8 d promotes cellular senescence, characterized by increased SA-β-gal activity, SASP, and p53/p21 expression [84,85,86].

Mitoxantrone is an anthracycline derivative developed to retain antitumor efficacy while reducing cardiotoxicity relative to doxorubicin [87,88]. It is widely used to treat several malignancies, including acute myeloid leukemia [87]. By inhibiting topoisomerase II, mitoxantrone disrupts cell-cycle progression and induces apoptosis in cancer cells [87]. At 2 µM for 24 h, mitoxantrone induces ICD in MCA205 fibrosarcoma cells by promoting CRT translocation and ATP release [89], with similar effects observed in H1299 and A549 lung cancer cells treated with 1 µM for 24 h [90]. In contrast, lower-dose treatment with 2 nM for 48–72 h induces senescence in A549 cells, characterized by enhanced SASP activity and increased p21 and p16 expression [91,92].

Cyclophosphamide is a widely used antineoplastic agent for treating hematologic malignancies, including non-Hodgkin lymphoma, and solid tumors such as breast and ovarian cancers [93]. At 20 µM for 4 h, cyclophosphamide induces ICD in GL261 and CT-2A glioma cells by promoting type-I interferon responses and DAMP release through ER stress and reactive oxygen species (ROS) generation [94]. At lower concentrations, it can also induce cellular senescence. In human fetal lung fibroblasts (TIG-7), treatment with 10 µM cyclophosphamide for 12 d activates MAP kinase signaling and promotes cellular senescence [95].

Bortezomib, a dipeptide boronic acid derivative, is the first FDA-approved proteasome inhibitor and prevents the degradation of misfolded and damaged proteins [96,97]. It exhibits antitumor activity in multiple myeloma, prostate cancer, breast cancer, and gastric cancer [97,98,99]. Bortezomib induces ICD by promoting CRT exposure and type 1 interferon responses in multiple myeloma, as demonstrated in AM01, H929, and 5TGM1 cells treated with 5–10 nM for 16 h and in U266 cells treated with 100 nM for 24 h [100,101,102]. At lower concentrations, bortezomib can induce cellular senescence; in A549 lung cancer cells, treatment with 25–50 nM for 14 d promotes telomere shortening and increases SA-β-gal expression [103].

Paclitaxel, an FDA-approved chemotherapeutic agent originally isolated from the yew tree, promotes microtubule assembly while inhibiting depolymerization, thereby disrupting cell division [104,105,106]. Through microtubule stabilization, cell-cycle arrest, and apoptosis induction, paclitaxel is effective against various cancers, including lung, breast, ovarian, and liver cancers, as well as those resistant to leukopenia [104,105,106]. At 10 µM for 48 h, paclitaxel induces ICD in ID8 ovarian cancer cells by increasing CRT, ATP, HMGB1, and ERp57 levels [107]. In contrast, treatment of high-grade serous ovarian cancer cells with a lower concentration of 25 nM for 72 h promotes senescence, as demonstrated by increased SA-β-gal activity and elevated p16, p21, p53, and SASP factors [108].

5-Fluorouracil is a widely used fluoropyrimidine chemotherapeutic agent for colorectal, breast, and gastrointestinal cancers. It inhibits thymidylate synthase, thereby suppressing S phase DNA replication and disrupting RNA processing [109,110,111]. At 800 µM for 24 h, 5-fluorouracil induces ICD in CT26 and HCT116 colon cancer cells by promoting DAMP release, including ATP and HMGB, and inactivating STAT3, thereby enhancing antitumor activity [112]. In contrast, lower concentrations promote cancer cell senescence; in RKO colon cancer cells, treatment with 10 µM for 12 or 24 h increases ROS generation, SA-β-gal activity, and SASP production [113].

Dactinomycin, also known as actinomycin D, is a natural antibiotic and transcriptional inhibitor that binds DNA [114]. It induces tumor cell apoptosis by disrupting anti-apoptotic gene expression and has demonstrated in vitro efficacy against osteosarcoma [115]. In U2OS osteosarcoma cells, dactinomycin induces ICD at 0.5–1 µM for 6–12 h, as evidenced by eIF2α phosphorylation, ATP and HMGB1 release, CRT exposure, and induction of type 1 interferon-related genes; similar effects occur in MCA205 fibrosarcoma cells treated with 0.5–2 µM for 6 h [116]. Conversely, at lower concentrations, dactinomycin can induce premature senescence, as shown by increased SA-β-gal activity and elevated SASP levels in AML3 acute myeloid leukemia cells treated with 5 nM for 2 h [117].

Oxaliplatin is a platinum-based chemotherapeutic agent widely used to treat colorectal, gastric, ovarian, pancreatic, and lung cancers [118]. Its cytotoxic activity is mediated by DNA damage, including inhibition of DNA synthesis and formation of DNA lesions, as well as disruption of messenger RNA synthesis through interactions with transcription factors and inhibition of RNA polymerase [119]. At 20 µM for 24 h, oxaliplatin induces ICD in LLC and KLN205 lung cancer cells through CRT exposure and HMGB1 and ATP release [120]; similarly, treatment of CT26 colon cancer cells with 5 µM for 4 h promotes DAMP release and eIF2α phosphorylation [121]. In contrast, lower-dose treatment with 0.5 µM for 5 d promotes cellular senescence in LoVo colon cancer cells by increasing SASP and senescent-cell anti-apoptotic pathway (SCAP) levels and activating the p53 and p21 pathways [122].

Resveratrol, a non-flavonoid polyphenol, exhibits anti-inflammatory, antioxidant, and antineoplastic activities [123,124,125]. It promotes cancer cell apoptosis and suppresses proliferation by inducing S-phase cell-cycle arrest and activating p53 and p21 [123]. At 25–50 µM for 24 h, resveratrol induces ICD in SKOV3, A2780, and ID8 ovarian cancer cells through CRT exposure and HMGB1 and ATP release [126]. Conversely, at lower concentrations, resveratrol can induce cellular senescence by reducing SIRT1/2 expression and increasing SASP levels. These effects have been observed in BJ fibroblasts treated with 10–100 µM for 72 h, U2OS osteosarcoma and A549 lung cancer cells treated with 50 µM for 7 d, and HT1080 fibrosarcoma cells treated with 25 µM for 7 d [127,128].

Docetaxel, a second-generation taxane antineoplastic agent, induces G2/M cell-cycle arrest by inhibiting microtubule depolymerization, thereby promoting cancer cell death, while increasing p27 expression and suppressing Bcl-2 [129,130]. In A549, NCI-H1975, and NCI-H1650 lung cancer cells, treatment with 0.5 µM docetaxel for 24–48 h induces ICD, characterized by CRT exposure, HMGB1 and ATP release, and enhanced phagocytosis and DC maturation [131]. At lower concentrations, however, docetaxel promotes cellular senescence; in A549 and NCI-H1299 lung cancer cells, treatment with 100 nM for 24 h followed by a 3 d recovery period inhibits DNA synthesis and increases IL-1β expression and SASP activity [132].

Wogonin, a monoflavonoid extracted from the Chinese medicinal plant *Scutellaria baicalensis*, exhibits notable anticancer activity [133,134]. It promotes apoptosis through p53 activation and induces cell-cycle arrest by inhibiting cyclins and CDKs in malignant cells [133,134]. At 100 µM for 2–4 h, wogonin induces ICD in MFC gastric carcinoma cells through translocation of Annexin A1 and CRT to the plasma membrane and release of ATP and HMGB1 [135]. Conversely, prolonged treatment with 100 µM for 8 d promotes cellular senescence in MDA-MB-231 breast cancer cells, characterized by increased p16 expression, enhanced β-galactosidase activity, and modulation of the SASP [136].

Shikonin, a natural flavonoid extracted from *Lithospermum erythrorhizon* and used in traditional Chinese medicine, exhibits antibacterial, antimicrobial, anti-inflammatory, and antineoplastic properties [137,138,139]. Its anticancer activity involves multiple mechanisms, including cell-cycle arrest, oxidative stress, autophagy, apoptosis, and necroptosis [137]. Shikonin induces ICD by upregulating DAMPs, including GRP78, CRT, HSP70, and HMGB1, in B16 melanoma cells treated with 5–20 µM for 24 h [140], CT26 colon cancer cells treated with 10 µM for 24 h [141], and 4T1 breast cancer cells treated with 5 µM for 24 h [142]. At lower concentrations, shikonin also promotes cellular senescence by inhibiting CDK4/6, activating the p53/p21/p16 pathway, and enhancing SASP and senescence-associated heterochromatin foci (SAHF) activity, as demonstrated in A549 lung cancer cells treated with 1–2 µM for 72 h and H1299 lung cancer cells treated with 0.3–0.6 µM for 72 h [143]. In addition, shikonin induces senescence in HT29 and HCT116 colon cancer cells at 4.31 and 3.39 µM, respectively, for 24 h by suppressing CDKN2A and CXCL8 and enhancing SA-β-Gal activity [144].

### 4.2. Dual Roles of ICD-Related Drugs in Modulating Immune Responses

Lyman [145], Odaimi et al. [146], and Lim et al. [147] reported that high-dose chemotherapy can elicit effective anti-tumor responses and may improve survival in patients with advanced cancer. High-dose chemotherapy may also help overcome drug resistance and benefit patients with poor prognoses, although its clinical utility is constrained by adverse effects, particularly cytotoxicity [148]. Many chemotherapeutic ICD inducers are first-generation anticancer agents designed to achieve extensive tumor destruction but can also damage normal tissues, limiting their suitability for patients with early-stage disease. In contrast, low-dose therapy can induce cellular senescence through sustained suppression of cell proliferation, potentially reducing chronic treatment-related toxicity while supporting long-term survival [70,149]. Moreover, Majumder et al. [150] and Choi et al. [151] reported that approximately 57% of neoplastic or early-stage prostate cancer specimens exhibited a senescent phenotype, suggesting that senescence-inducing strategies may have therapeutic potential in premalignant or early-stage cancers. Understanding these dose- and time-dependent dual effects—ICD versus senescence—may therefore enable optimization of anticancer therapy to maximize efficacy while minimizing toxicity.

**Figure 4:**
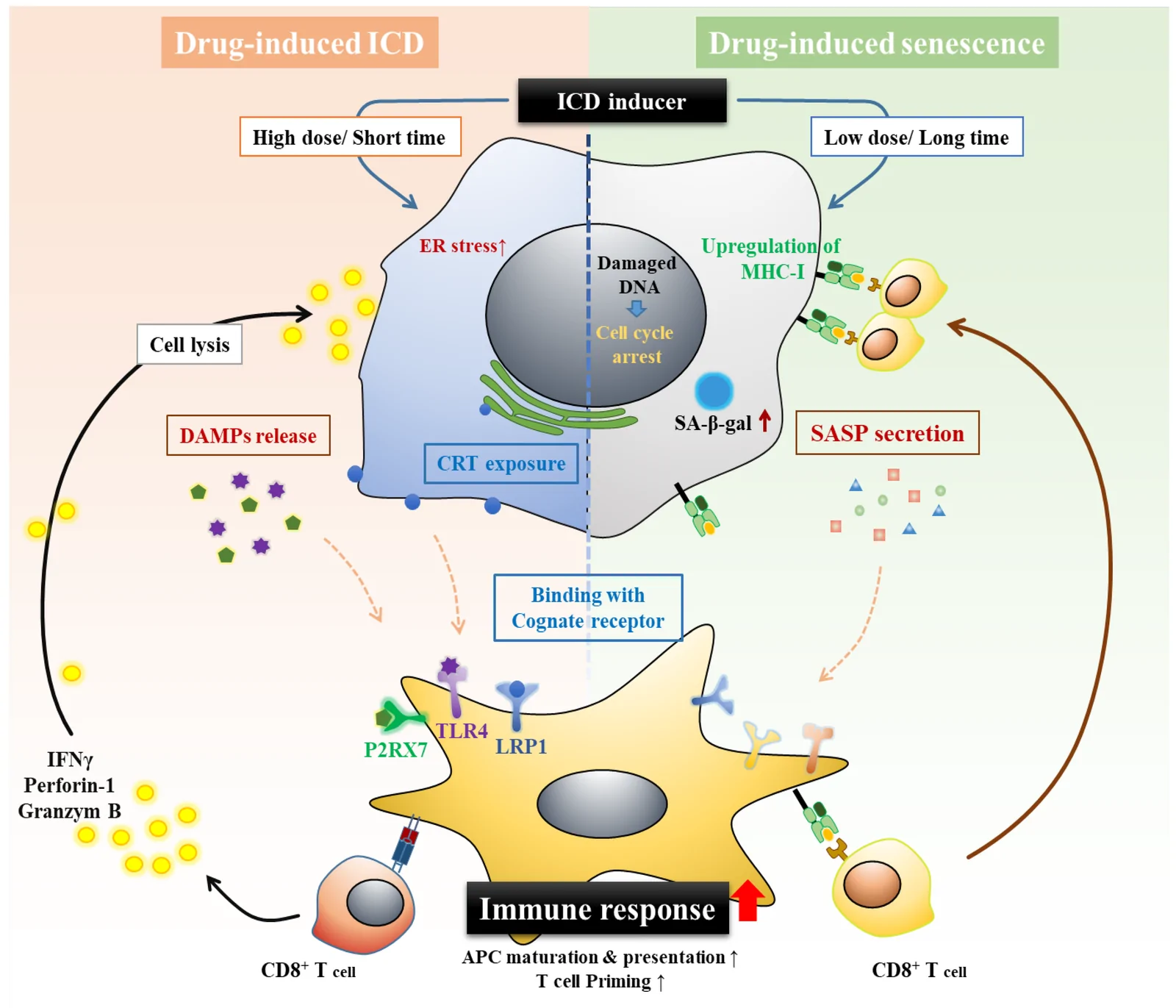
Immunostimulatory effects of drug-induced ICD and senescence. ICD-inducing chemotherapeutic agents can elicit distinct cellular stress responses depending on dose and treatment duration. High-dose, short-term treatment with agents such as doxorubicin induces ICD, whereas lower-dose, prolonged exposure to the same agents can promote drug-induced senescence. Although these responses differ fundamentally—cell death versus cell-cycle arrest—both can stimulate T-cell-mediated immune responses and enhance antitumor effects.

As summarized in Table 1, the same agents can induce ICD under high-dose, short-duration conditions or senescence under low-dose, prolonged treatment. However, there are limitations to interpreting these findings comprehensively as a concentration- and time-dependent relationship. The summarized studies were conducted in different cancer cell types, under different treatment schedules and experimental conditions, and with different readouts; they therefore do not represent direct comparisons of ICD and senescence under matched experimental settings. As shown in Table 1, resveratrol and wogonin have been reported to induce either ICD or senescence at identical or overlapping concentrations, but these observations were obtained in different cell types and under different experimental conditions. Such differences should be considered when interpreting whether a given agent preferentially induces ICD or senescence. Furthermore, as noted in Section 2, the ICD classification in Table 1 is based primarily on molecular hallmarks such as CRT exposure and DAMP release. Functional confirmation, including dendritic cell engulfment and maturation as well as downstream T-cell recruitment and activation, has been reported for only a subset of these agents, and the immunogenic potential of the remaining agents should therefore be interpreted with caution. Within these limits, the available data nevertheless suggest that lower-dose administration of established ICD-inducing agents may, in certain experimental settings, promote drug-induced senescence and SASP-associated pro-inflammatory signaling, thereby contributing to antitumor immune responses, including CD8^+^ T-cell activation. (Figure 4). Although ICD and senescence differ fundamentally in their physiological outcomes—cytotoxic cell death versus stable growth arrest—both can enhance antitumor immunity and may therefore synergize with immunotherapies, including anti-PD-1/PD-L1 and anti-CTLA-4 treatments. Although combinations of ICIs with ICD or senescence inducers remain at an early stage of investigation, these strategies have substantial potential as adjunctive approaches to cancer immunotherapy.

## 5. Conclusions

While early anticancer strategies primarily focused on direct tumor cell elimination, recent approaches increasingly harness the host immune system. Although immunotherapies, including ICIs (anti-PD-1/PD-L1 and anti-CTLA-4), can achieve substantial antitumor effects, their efficacy remains limited in patients with a non-immunogenic TME. Accordingly, strategies that convert immunologically ‘cold’ tumors into ‘hot’ tumors are needed to improve therapeutic responses. ICD represents one such approach, and considerable efforts are underway to develop new or modified compounds capable of inducing ICD. However, the inherent cytotoxicity of many ICD-inducing agents remains a major limitation to their broad application in combination with immunotherapy. Drug-induced senescence represents an alternative strategy for enhancing tumor immunogenicity. Notably, established ICD inducers can promote cytotoxic ICD at high concentrations but induce growth-arresting cellular senescence at lower concentrations (Table 1). Unlike ICD, drug-induced senescence does not directly cause cytotoxic cell death but can nevertheless stimulate immune responses through stable cell-cycle arrest and the release of inflammatory mediators associated with the SASP. Collectively, the evidence reviewed here suggests that the induction of ICD or senescence by the same agent reflects an interplay between drug concentration, treatment duration, and cell- or model-specific susceptibility. Because the studies summarized in Table 1 were performed in different cell types and under different treatment schedules, this relationship should be regarded as a general trend rather than a universal rule. Within these limits, a broadly similar pattern was noted across several agents and tumor models, in which shorter exposure to higher concentrations was more often associated with ICD, whereas prolonged exposure to lower, sublethal concentrations was more often associated with senescence. These observations raise the possibility that therapeutic dose and exposure time might be tailored according to the patient’s condition and tumor stage, although this would require validation under matched experimental conditions in models reflecting the intended tumor context. In early-stage disease, particularly when drug toxicity is a major concern, lower-dose or prolonged treatment designed to favor senescence may reduce damage to normal tissues. Conversely, in advanced or terminal-stage malignancies, treatment strategies favoring ICD may be considered to combine direct tumor cytotoxicity with immune activation.

Overall, drug-induced ICD and senescence represent complementary strategies with the potential to synergize with cancer immunotherapy. The evidence summarized in this review may provide a starting point for considering how these distinct cellular responses could be applied according to patient characteristics and tumor stage. This concept remains preliminary, however, as it is derived from studies performed under heterogeneous experimental conditions and largely in vitro, and because the same agent may elicit different outcomes depending on the cell type and model used. Systematic comparisons under matched experimental settings, together with validation in immunocompetent in vivo models, will therefore be required before such an approach can be considered for clinical application. With such validation, continued research and therapeutic innovation may enable more precise exploitation of the ICD–senescence axis, ultimately providing new opportunities to optimize cancer immunotherapy and improve patient outcomes.

## Figures and Tables

**Table 1:** Dose- and time-dependent induction of immunogenic cell death (ICD) and senescence by ICD inducers.

Drug Name	Type	Dose & Time	Mechanism	Ref.
Doxorubicin	ICD	25 µM for 24 h	Elicits an antitumor immune response through expression of endogenous adjuvants (HSP70 and HMGB1) and enhances cytotoxic CD8^+^ T-cell infiltration	[81,82,83]
	Senescence	0.1 µM for 4~8 d	Increases p53, p21, and cyclin D1 expression	[84,85,86]
Mitoxantrone	ICD	1~2 µM for 24 h	Promotes CRT translocation and ATP release	[89,90]
	Senescence	2 nM for 48~72 h	Enhances SASP activity and increases p21 and p16 expression	[91,92]
Cyclophosphamide	ICD	20 µM for 4 h	Enhances type-I interferon signaling and DAMP release through ER stress and ROS generation	[94]
	Senescence	10 µM for 12 d	Activates the MAP kinase signaling pathway	[95]
Bortezomib	ICD	5~10 or 100 nM for 16 or 24 h	Promotes CRT exposure and enhances type-1 interferon responses	[100,101,102]
	Senescence	25~50 nM for 14 d	Increases SA-β-galactosidase expression and induces telomere shortening	[103]
Paclitaxel	ICD	10 µM for 48 h	Increases CRT, ATP, HMGB1, and ERp57 levels	[107]
	Senescence	25 nM for 72 h	Increases SA-β-gal activity and elevates p16, p21, p53, and SASP factors	[108]
5-fluorouracil	ICD	800 µM for 24 h	Induces ATP and HMGB release, accompanied by STAT3 inactivation	[112]
	Senescence	10 µM for 12 or 24 h	Increases ROS generation, SA-β-gal activity, and SASP release	[113]
Dactinomycin	ICD	0.5~2 µM for 6 or 12 h	Induces eIF2α phosphorylation, ATP and HMGB1 release, CRT exposure, and type 1 interferon-related gene expression	[116]
	Senescence	5 nM for 2 h	Increases SA-β-gal activity and SASP	[117]
Oxaliplatin	ICD	5 µM for 4 h	Promotes HMGB1 and ATP release and increases eIF2α phosphorylation	[121]
	Senescence	0.5 µM for 5 d	Elevates SASP and SCAP levels and activates the p53 and p21 pathways	[122]
Resveratrol	ICD	25–50 µM for 24 h	Promotes CRT exposure and HMGB1 and ATP release	[126]
	Senescence	10~100 µM for 3 or 7 d	Reduces SIRT1/2 expression and increases SASP levels	[127,128]
Docetaxel	ICD	0.5 µM for 24–48 h	Promotes CRT exposure and HMGB1/ATP release Enhances phagocytosis and DC maturation	[131]
	Senescence	100 nM for 24 h + 3 d (recovery period)	Inhibits DNA synthesis and increases IL-1β expression and SASP activity	[132]
Wogonin	ICD	100 µM for 2 or 4 h	Promotes Annexin A1 and CRT translocation to the plasma membrane and ATP and HMGB1 release	[135]
	Senescence	100 µM for 8 d	Increases p16 expression and β-galactosidase activity and modulates the SASP	[136]
Shikonin	ICD	5~20 µM for 24 h	Upregulates GRP78, CRT, HSP70, and HMGB1 expression	[140,141,142]
	Senescence	0.3~2 µM for 72 h 4.31 and 3.39 µM for 24 h	Inhibits CDK4/6, activates the p53/p21/p16 pathway, and enhances SASP and SAHF activity Suppresses CDKN2A and CXCL8 and enhances SA-β-Gal activity	[143,144]

Abbreviations: CRT, calreticulin; DAMP, damage-associated molecular patterns; ER, endoplasmic reticulum; ICD, immunogenic cell death; ROS, reactive oxygen species; SASP, senescence-associated secretory phenotype; SCAP, senescent-cell anti-apoptotic pathway; HSP70, heat shock proteins 70; GRP78, glucose-regulated protein 78; HMGB1, high mobility group box 1; SAHF, senescence-associated heterochromatin foci.

## Data Availability

No new data were created in this study. Data sharing is not applicable to this article.

## Abbreviations

The following abbreviations are used in this manuscript:

APCs	antigen-presenting cells
ATF4	activating transcription factor 4
ATF6	activating transcription factor 6
Bak	Bcl-2 homologous antagonist/killer
BAP31	B-cell receptor-associated protein 31
Bax	BCL2-associated X protein
CDKs	cyclin-dependent kinases
cGAMP	cGAS synthesizes cyclic GMP-AMP
cGAS	Cyclic GMP-AMP synthase
CHOP	C/enhancer binding protein-homologous protein
CRT	Calreticulin
DAMPs	damage-associated molecular patterns
DCs	dendritic cells
dsDNA	double-stranded DNA
eIF2α	eukaryotic translation initiation factor 2α
ER	endoplasmic reticulum
HMGB1	high mobility group box 1
ICD	immunogenic cell death
ICIs	immune checkpoint inhibitors
IKK	IκB kinase
IRE1α	inositol-requiring enzyme 1α
LRP1	low-density lipoprotein receptor-related protein 1
MHC	major histocompatibility complex class
NF-κB	nuclear factor-κB
PERK	protein kinase RNA-like endoplasmic reticulum kinase
PRRs	pattern-recognition receptors
SASP	senescence-associated secretory phenotype
SAHF	senescence-associated heterochromatin foci
SCAP	senescent-cell anti-apoptotic pathway
TBK1	TANK-binding kinase 1
TLR	toll-like receptor
TME	Tumor microenvironment

## References

[1] Bray F., Laversanne M., Weiderpass E., Soerjomataram I. (2021). The ever-increasing importance of cancer as a leading cause of premature death worldwide. Cancer.

[2] Bray F., Laversanne M., Sung H., Ferlay J., Siegel R.L., Soerjomataram I., Jemal A. (2024). Global cancer statistics 2022: GLOBOCAN estimates of incidence and mortality worldwide for 36 cancers in 185 countries. CA Cancer J. Clin..

[3] Liu B., Zhou H., Tan L., Siu K.T.H., Guan X.Y. (2024). Exploring treatment options in cancer: Tumor treatment strategies. Signal Transduct. Target. Ther..

[4] Bedard P.L., Hyman D.M., Davids M.S., Siu L.L. (2020). Small molecules, big impact: 20 years of targeted therapy in oncology. Lancet.

[5] Zhang Y., Zhang Z. (2020). The history and advances in cancer immunotherapy: Understanding the characteristics of tumor-infiltrating immune cells and their therapeutic implications. Cell Mol. Immunol..

[6] Liu C., Yang M., Zhang D., Chen M., Zhu D. (2022). Clinical cancer immunotherapy: Current progress and prospects. Front. Immunol..

[7] Galon J., Bruni D. (2019). Approaches to treat immune hot, altered and cold tumours with combination immunotherapies. Nat. Rev. Drug Discov..

[8] Wang L., Geng H., Liu Y., Liu L., Chen Y., Wu F., Liu Z., Ling S., Wang Y., Zhou L. (2023). Hot and cold tumors: Immunological features and the therapeutic strategies. MedComm.

[9] Bonaventura P., Shekarian T., Alcazer V., Valladeau-Guilemond J., Valsesia-Wittmann S., Amigorena S., Caux C., Depil S. (2019). Cold Tumors: A Therapeutic Challenge for Immunotherapy. Front. Immunol..

[10] Duan Q., Zhang H., Zheng J., Zhang L. (2020). Turning Cold into Hot: Firing up the Tumor Microenvironment. Trends Cancer.

[11] Wu B., Zhang B., Li B., Wu H., Jiang M. (2024). Cold and hot tumors: From molecular mechanisms to targeted therapy. Signal Transduct. Target. Ther..

[12] Wei C., Ma Y., Wang F., Liao Y., Chen Y., Zhao B., Zhao Q., Wang D., Tang D. (2022). Igniting Hope for Tumor Immunotherapy: Promoting the “Hot and Cold” Tumor Transition. Clin. Med. Insights Oncol..

[13] Fucikova J., Kepp O., Kasikova L., Petroni G., Yamazaki T., Liu P., Zhao L., Spisek R., Kroemer G., Galluzzi L. (2020). Detection of immunogenic cell death and its relevance for cancer therapy. Cell Death Dis..

[14] MacNabb B.W., Tumuluru S., Chen X., Godfrey J., Kasal D.N., Yu J., Jongsma M.L.M., Spaapen R.M., Kline D.E., Kline J. (2022). Dendritic cells can prime anti-tumor CD8^+^ T cell responses through major histocompatibility complex cross-dressing. Immunity.

[15] Liu Y.T., Sun Z.J. (2021). Turning cold tumors into hot tumors by improving T-cell infiltration. Theranostics.

[16] Lee M., Lee J.S. (2014). Exploiting tumor cell senescence in anticancer therapy. BMB Rep..

[17] Marin I., Boix O., Garcia-Garijo A., Sirois I., Caballe A., Zarzuela E., Ruano I., Attolini C.S., Prats N., Lopez-Dominguez J.A. (2023). Cellular Senescence Is Immunogenic and Promotes Antitumor Immunity. Cancer Discov..

[18] Bezu L., Gomes-da-Silva L.C., Dewitte H., Breckpot K., Fucikova J., Spisek R., Galluzzi L., Kepp O., Kroemer G. (2015). Combinatorial strategies for the induction of immunogenic cell death. Front. Immunol..

[19] Wang Y.-J., Fletcher R., Yu J., Zhang L. (2018). Immunogenic effects of chemotherapy-induced tumor cell death. Genes Dis..

[20] Kawano M., Tanaka K., Itonaga I., Iwasaki T., Miyazaki M., Ikeda S., Tsumura H. (2016). Dendritic cells combined with doxorubicin induces immunogenic cell death and exhibits antitumor effects for osteosarcoma. Oncol. Lett..

[21] Turubanova V.D., Balalaeva I.V., Mishchenko T.A., Catanzaro E., Alzeibak R., Peskova N.N., Efimova I., Bachert C., Mitroshina E.V., Krysko O. (2019). Immunogenic cell death induced by a new photodynamic therapy based on photosens and photodithazine. J. Immunother. Cancer.

[22] Zhu M., Yang M., Zhang J., Yin Y., Fan X., Zhang Y., Qin S., Zhang H., Yu F. (2021). Immunogenic cell death induction by ionizing radiation. Front. Immunol..

[23] Choi M., Shin J., Lee C.E., Chung J.Y., Kim M., Yan X., Yang W.H., Cha J.H. (2023). Immunogenic cell death in cancer immunotherapy. BMB Rep..

[24] Krysko D.V., Garg A.D., Kaczmarek A., Krysko O., Agostinis P., Vandenabeele P. (2012). Immunogenic cell death and DAMPs in cancer therapy. Nat. Rev. Cancer.

[25] Kroemer G., Galassi C., Zitvogel L., Galluzzi L. (2022). Immunogenic cell stress and death. Nat. Immunol..

[26] Kielbik M., Szulc-Kielbik I., Klink M. (2021). Calreticulin—Multifunctional chaperone in immunogenic cell death: Potential significance as a prognostic biomarker in ovarian cancer patients. Cells.

[27] Chaput N., De Botton S., Obeid M., Apetoh L., Ghiringhelli F., Panaretakis T., Flament C., Zitvogel L., Kroemer G. (2007). Molecular determinants of immunogenic cell death: Surface exposure of calreticulin makes the difference. J. Mol. Med..

[28] Garg A.D., Romano E., Rufo N., Agostinis P. (2016). Immunogenic versus tolerogenic phagocytosis during anticancer therapy: Mechanisms and clinical translation. Cell Death Differ..

[29] Obeid M., Tesniere A., Ghiringhelli F., Fimia G.M., Apetoh L., Perfettini J.L., Castedo M., Mignot G., Panaretakis T., Casares N. (2007). Calreticulin exposure dictates the immunogenicity of cancer cell death. Nat. Med..

[30] Ahmed A., Tait S.W. (2020). Targeting immunogenic cell death in cancer. Mol. Oncol..

[31] Gupta G., Borglum K., Chen H. (2021). Immunogenic cell death: A step ahead of autophagy in cancer therapy. J. Cancer Immunol..

[32] Ghiringhelli F., Apetoh L., Tesniere A., Aymeric L., Ma Y., Ortiz C., Vermaelen K., Panaretakis T., Mignot G., Ullrich E. (2009). Activation of the NLRP3 inflammasome in dendritic cells induces IL-1beta-dependent adaptive immunity against tumors. Nat. Med..

[33] Serrano-del Valle A., Anel A., Naval J., Marzo I. (2019). Immunogenic cell death and immunotherapy of multiple myeloma. Front. Cell Dev. Biol..

[34] Apetoh L., Ghiringhelli F., Tesniere A., Obeid M., Ortiz C., Criollo A., Mignot G., Maiuri M.C., Ullrich E., Saulnier P. (2007). Toll-like receptor 4–dependent contribution of the immune system to anticancer chemotherapy and radiotherapy. Nat. Med..

[35] Nayagom B., Amara I., Habiballah M., Amrouche F., Beaune P., de Waziers I. (2019). Immunogenic cell death in a combined synergic gene-and immune-therapy against cancer. Oncoimmunology.

[36] Galluzzi L., Kepp O., Hett E., Kroemer G., Marincola F.M. (2023). Immunogenic cell death in cancer: Concept and therapeutic implications. J. Transl. Med..

[37] Galluzzi L., Vitale I., Warren S., Adjemian S., Agostinis P., Martinez A.B., Chan T.A., Coukos G., Demaria S., Deutsch E. (2020). Consensus guidelines for the definition, detection and interpretation of immunogenic cell death. J. Immunother. Cancer.

[38] Collett G.P., Redman C.W., Sargent I.L., Vatish M. (2018). Endoplasmic reticulum stress stimulates the release of extracellular vesicles carrying danger-associated molecular pattern (DAMP) molecules. Oncotarget.

[39] Gong T., Liu L., Jiang W., Zhou R. (2020). DAMP-sensing receptors in sterile inflammation and inflammatory diseases. Nat. Rev. Immunol..

[40] Iurlaro R., Munoz-Pinedo C. (2016). Cell death induced by endoplasmic reticulum stress. FEBS J..

[41] Panaretakis T., Kepp O., Brockmeier U., Tesniere A., Bjorklund A.C., Chapman D.C., Durchschlag M., Joza N., Pierron G., van Endert P. (2009). Mechanisms of pre-apoptotic calreticulin exposure in immunogenic cell death. EMBO J..

[42] Rozpedek W., Pytel D., Mucha B., Leszczynska H., Diehl J.A., Majsterek I. (2016). The Role of the PERK/eIF2alpha/ATF4/CHOP Signaling Pathway in Tumor Progression During Endoplasmic Reticulum Stress. Curr. Mol. Med..

[43] Klymenko O., Huehn M., Wilhelm J., Wasnick R., Shalashova I., Ruppert C., Henneke I., Hezel S., Guenther K., Mahavadi P. (2019). Regulation and role of the ER stress transcription factor CHOP in alveolar epithelial type-II cells. J. Mol. Med..

[44] Fu X., Cui J., Meng X., Jiang P., Zheng Q., Zhao W., Chen X. (2021). Endoplasmic reticulum stress, cell death and tumor: Association between endoplasmic reticulum stress and the apoptosis pathway in tumors. Oncol. Rep..

[45] Rapoport B.L., Anderson R. (2019). Realizing the clinical potential of immunogenic cell death in cancer chemotherapy and radiotherapy. Int. J. Mol. Sci..

[46] Lin R.A., Lin J.K., Lin S.Y. (2021). Mechanisms of immunogenic cell death and immune checkpoint blockade therapy. Kaohsiung J. Med. Sci..

[47] Galluzzi L., Buque A., Kepp O., Zitvogel L., Kroemer G. (2017). Immunogenic cell death in cancer and infectious disease. Nat. Rev. Immunol..

[48] Amjad M.T., Chidharla A., Kasi A. (2024). Cancer Chemotherapy. StatPearls.

[49] Xie D., Wang Q., Wu G. (2022). Research progress in inducing immunogenic cell death of tumor cells. Front. Immunol..

[50] Faheem M.M., Seligson N.D., Ahmad S.M., Rasool R.U., Gandhi S.G., Bhagat M., Goswami A. (2020). Convergence of therapy-induced senescence (TIS) and EMT in multistep carcinogenesis: Current opinions and emerging perspectives. Cell Death Discov..

[51] Schmitt C.A., Wang B., Demaria M. (2022). Senescence and cancer—Role and therapeutic opportunities. Nat. Rev. Clin. Oncol..

[52] Kumari R., Jat P. (2021). Mechanisms of Cellular Senescence: Cell Cycle Arrest and Senescence Associated Secretory Phenotype. Front. Cell Dev. Biol..

[53] Takahashi A., Ohtani N., Yamakoshi K., Iida S., Tahara H., Nakayama K., Nakayama K.I., Ide T., Saya H., Hara E. (2006). Mitogenic signalling and the p16INK4a-Rb pathway cooperate to enforce irreversible cellular senescence. Nat. Cell Biol..

[54] Rovillain E., Mansfield L., Lord C.J., Ashworth A., Jat P.S. (2011). An RNA interference screen for identifying downstream effectors of the p53 and pRB tumour suppressor pathways involved in senescence. BMC Genom..

[55] Bousset L., Gil J. (2022). Targeting senescence as an anticancer therapy. Mol. Oncol..

[56] Wang L., Lankhorst L., Bernards R. (2022). Exploiting senescence for the treatment of cancer. Nat. Rev. Cancer.

[57] Chibaya L., Snyder J., Ruscetti M. (2022). Senescence and the tumor-immune landscape: Implications for cancer immunotherapy. Semin. Cancer Biol..

[58] Sagiv A., Krizhanovsky V. (2013). Immunosurveillance of senescent cells: The bright side of the senescence program. Biogerontology.

[59] Ouvrier B., Ismael S., Bix G.J. (2024). Senescence and SASP Are Potential Therapeutic Targets for Ischemic Stroke. Pharmaceuticals.

[60] Ohtani N. (2022). The roles and mechanisms of senescence-associated secretory phenotype (SASP): Can it be controlled by senolysis?. Inflamm. Regen..

[61] Coppé J.-P., Desprez P.-Y., Krtolica A., Campisi J. (2010). The senescence-associated secretory phenotype: The dark side of tumor suppression. Annu. Rev. Pathol. Mech. Dis..

[62] Demirci D., Dayanc B., Mazi F.A., Senturk S. (2021). The Jekyll and Hyde of cellular senescence in cancer. Cells.

[63] Cuollo L., Antonangeli F., Santoni A., Soriani A. (2020). The senescence-associated secretory phenotype (SASP) in the challenging future of cancer therapy and age-related diseases. Biology.

[64] Gulen M.F., Samson N., Keller A., Schwabenland M., Liu C., Glück S., Thacker V.V., Favre L., Mangeat B., Kroese L.J. (2023). cGAS–STING drives ageing-related inflammation and neurodegeneration. Nature.

[65] Schmitz C.R.R., Maurmann R.M., Guma F.T., Bauer M.E., Barbe-Tuana F.M. (2023). cGAS-STING pathway as a potential trigger of immunosenescence and inflammaging. Front. Immunol..

[66] Zheng W., Feng D., Xiong X., Liao X., Wang S., Xu H., Le W., Wei Q., Yang L. (2023). The role of cGAS-STING in age-related diseases from mechanisms to therapies. Aging Dis..

[67] Loo T.M., Miyata K., Tanaka Y., Takahashi A. (2020). Cellular senescence and senescence-associated secretory phenotype via the cGAS-STING signaling pathway in cancer. Cancer Sci..

[68] Zhang D., Zhang J.-W., Xu H., Chen X., Gao Y., Jiang H.-G., Wang Y., Wu H., Yang L., Wang W.-B. (2024). Therapy-induced senescent tumor cell-derived extracellular vesicles promote colorectal cancer progression through SERPINE1-mediated NF-κB p65 nuclear translocation. Mol. Cancer.

[69] Yang J., Liu M., Hong D., Zeng M., Zhang X. (2021). The paradoxical role of cellular senescence in cancer. Front. Cell Dev. Biol..

[70] Prasanna P.G., Citrin D.E., Hildesheim J., Ahmed M.M., Venkatachalam S., Riscuta G., Xi D., Zheng G., Deursen J.v., Goronzy J. (2021). Therapy-induced senescence: Opportunities to improve anticancer therapy. J. Natl. Cancer Inst..

[71] Hinds P., Pietruska J. (2017). Senescence and tumor suppression. F1000Research.

[72] Liu Y., Lomeli I., Kron S.J. (2024). Therapy-Induced Cellular Senescence: Potentiating Tumor Elimination or Driving Cancer Resistance and Recurrence?. Cells.

[73] Chambers C.R., Ritchie S., Pereira B.A., Timpson P. (2021). Overcoming the senescence-associated secretory phenotype (SASP): A complex mechanism of resistance in the treatment of cancer. Mol. Oncol..

[74] Liu K., Huang H., Zhang M., Chen S., Yang Y., Fang C., Zhong X. (2025). When therapy-induced senescence meets tumors: A double-edged sword: A review. Medicine.

[75] Takasugi M., Yoshida Y., Ohtani N. (2022). Cellular senescence and the tumour microenvironment. Mol. Oncol..

[76] Marin I., Serrano M., Pietrocola F. (2023). Cellular senescence enhances adaptive anticancer immunosurveillance. Oncoimmunology.

[77] Mattioli R., Ilari A., Colotti B., Mosca L., Fazi F., Colotti G. (2023). Doxorubicin and other anthracyclines in cancers: Activity, chemoresistance and its overcoming. Mol. Asp. Med..

[78] Thorn C.F., Oshiro C., Marsh S., Hernandez-Boussard T., McLeod H., Klein T.E., Altman R.B. (2011). Doxorubicin pathways: Pharmacodynamics and adverse effects. Pharmacogenet. Genom..

[79] Yang S.H., Kim J.S., Kim D.K., Yoon T.H., Cho M., Cho S.K. (2025). ACF-01 enhances the anticancer effect of doxorubicin by suppressing stemness and inducing apoptosis in triple-negative breast cancer stem-like cells. Appl. Biol. Chem..

[80] Linders A.N., Dias I.B., Lopez Fernandez T., Tocchetti C.G., Bomer N., Van der Meer P. (2024). A review of the pathophysiological mechanisms of doxorubicin-induced cardiotoxicity and aging. npj Aging.

[81] Vanmeerbeek I., Sprooten J., De Ruysscher D., Tejpar S., Vandenberghe P., Fucikova J., Spisek R., Zitvogel L., Kroemer G., Galluzzi L. (2020). Trial watch: Chemotherapy-induced immunogenic cell death in immuno-oncology. Oncoimmunology.

[82] Demaria M., O’Leary M.N., Chang J., Shao L., Liu S., Alimirah F., Koenig K., Le C., Mitin N., Deal A.M. (2017). Cellular Senescence Promotes Adverse Effects of Chemotherapy and Cancer Relapse. Cancer Discov..

[83] Casares N., Pequignot M.O., Tesniere A., Ghiringhelli F., Roux S., Chaput N., Schmitt E., Hamai A., Hervas-Stubbs S., Obeid M. (2005). Caspase-dependent immunogenicity of doxorubicin-induced tumor cell death. J. Exp. Med..

[84] Wang R., Sun L., Xia S., Wu H., Ma Y., Zhan S., Zhang G., Zhang X., Shi T., Chen W. (2021). B7-H3 suppresses doxorubicin-induced senescence-like growth arrest in colorectal cancer through the AKT/TM4SF1/SIRT1 pathway. Cell Death Dis..

[85] Wang Z., Li Y., Wu D., Yu S., Wang Y., Chan F.L. (2020). Correction: Nuclear receptor HNF4alpha performs a tumor suppressor function in prostate cancer via its induction of p21-driven cellular senescence. Oncogene.

[86] Sliwinska M.A., Mosieniak G., Wolanin K., Babik A., Piwocka K., Magalska A., Szczepanowska J., Fronk J., Sikora E. (2009). Induction of senescence with doxorubicin leads to increased genomic instability of HCT116 cells. Mech. Ageing Dev..

[87] Fox E.J. (2004). Mechanism of action of mitoxantrone. Neurology.

[88] Koeller J., Eble M. (1988). Mitoxantrone: A novel anthracycline derivative. Clin. Pharm..

[89] Sukkurwala A.Q., Adjemian S., Senovilla L., Michaud M., Spaggiari S., Vacchelli E., Baracco E.E., Galluzzi L., Zitvogel L., Kepp O. (2014). Screening of novel immunogenic cell death inducers within the NCI Mechanistic Diversity Set. Oncoimmunology.

[90] Valanciute A., Mathieson L., O’Connor R.A., Scott J.I., Vendrell M., Dorward D.A., Akram A.R., Dhaliwal K. (2022). Phototherapeutic Induction of Immunogenic Cell Death and CD8+ T Cell-Granzyme B Mediated Cytolysis in Human Lung Cancer Cells and Organoids. Cancers.

[91] Petrova N.V., Velichko A.K., Razin S.V., Kantidze O.L. (2016). Small molecule compounds that induce cellular senescence. Aging Cell.

[92] Zhao H., Halicka H.D., Traganos F., Jorgensen E., Darzynkiewicz Z. (2010). New biomarkers probing depth of cell senescence assessed by laser scanning cytometry. Cytom. A.

[93] Emadi A., Jones R.J., Brodsky R.A. (2009). Cyclophosphamide and cancer: Golden anniversary. Nat. Rev. Clin. Oncol..

[94] Du B., Waxman D.J. (2020). Medium dose intermittent cyclophosphamide induces immunogenic cell death and cancer cell autonomous type I interferon production in glioma models. Cancer Lett..

[95] Palaniyappan A. (2009). Cyclophosphamide induces premature senescence in normal human fibroblasts by activating MAP kinases. Biogerontology.

[96] Alwahsh M., Farhat J., Talhouni S., Hamadneh L., Hergenroder R. (2023). Bortezomib advanced mechanisms of action in multiple myeloma, solid and liquid tumors along with its novel therapeutic applications. EXCLI J..

[97] Orlowski R.Z., Stinchcombe T.E., Mitchell B.S., Shea T.C., Baldwin A.S., Stahl S., Adams J., Esseltine D.L., Elliott P.J., Pien C.S. (2002). Phase I trial of the proteasome inhibitor PS-341 in patients with refractory hematologic malignancies. J. Clin. Oncol..

[98] Adams J., Palombella V.J., Sausville E.A., Johnson J., Destree A., Lazarus D.D., Maas J., Pien C.S., Prakash S., Elliott P.J. (1999). Proteasome inhibitors: A novel class of potent and effective antitumor agents. Cancer Res..

[99] Ci X., Li B., Ma X., Kong F., Zheng C., Bjorkholm M., Jia J., Xu D. (2015). Bortezomib-mediated down-regulation of telomerase and disruption of telomere homeostasis contributes to apoptosis of malignant cells. Oncotarget.

[100] Zitvogel L., Kroemer G. (2021). Bortezomib Induces Immunogenic Cell Death in Multiple Myeloma. Blood Cancer Discov..

[101] Gulla A., Morelli E., Samur M.K., Botta C., Hideshima T., Bianchi G., Fulciniti M., Malvestiti S., Prabhala R.H., Talluri S. (2021). Bortezomib induces anti-multiple myeloma immune response mediated by cGAS/STING pathway activation. Blood Cancer Discov..

[102] Spisek R., Charalambous A., Mazumder A., Vesole D.H., Jagannath S., Dhodapkar M.V. (2007). Bortezomib enhances dendritic cell (DC)-mediated induction of immunity to human myeloma via exposure of cell surface heat shock protein 90 on dying tumor cells: Therapeutic implications. Blood.

[103] Wang L., Yin H., Huang S., Huang S., Huang C., Zhang Z., Liu H. (2022). Bortezomib induces cellular senescence in A549 lung cancer cells by stimulating telomere shortening. Hum. Exp. Toxicol..

[104] Lu J., Xu F., Rao C., Shen C., Jin J., Zhu Z., Wang C., Li Q. (2022). Mechanism of action of paclitaxel for treating glioblastoma based on single-cell RNA sequencing data and network pharmacology. Front. Pharmacol..

[105] Calaf G.M., Ponce-Cusi R., Carrion F. (2018). Curcumin and paclitaxel induce cell death in breast cancer cell lines. Oncol. Rep..

[106] Nawara H.M., Afify S.M., Hassan G., Zahra M.H., Seno A., Seno M. (2021). Paclitaxel-Based Chemotherapy Targeting Cancer Stem Cells from Mono- to Combination Therapy. Biomedicines.

[107] Lau T.S., Chan L.K.Y., Man G.C.W., Wong C.H., Lee J.H.S., Yim S.F., Cheung T.H., McNeish I.A., Kwong J. (2020). Paclitaxel Induces Immunogenic Cell Death in Ovarian Cancer via TLR4/IKK2/SNARE-Dependent Exocytosis. Cancer Immunol. Res..

[108] Uruski P., Sepetowska A., Konieczna C., Pakula M., Wyrwa M., Tussupkaliyev A., Tykarski A., Mikula-Pietrasik J., Ksiazek K. (2021). Primary high-grade serous ovarian cancer cells are sensitive to senescence induced by carboplatin and paclitaxel in vitro. Cell Mol. Biol. Lett..

[109] Longley D.B., Harkin D.P., Johnston P.G. (2003). 5-fluorouracil: Mechanisms of action and clinical strategies. Nat. Rev. Cancer.

[110] Ghafouri-Fard S., Abak A., Tondro Anamag F., Shoorei H., Fattahi F., Javadinia S.A., Basiri A., Taheri M. (2021). 5-Fluorouracil: A Narrative Review on the Role of Regulatory Mechanisms in Driving Resistance to This Chemotherapeutic Agent. Front. Oncol..

[111] Altieri P., Murialdo R., Barisione C., Lazzarini E., Garibaldi S., Fabbi P., Ruggeri C., Borile S., Carbone F., Armirotti A. (2017). 5-fluorouracil causes endothelial cell senescence: Potential protective role of glucagon-like peptide 1. Br. J. Pharmacol..

[112] Jie Y., Yang X., Chen W. (2022). Pulsatilla Decoction Combined with 5-Fluorouracil Triggers Immunogenic Cell Death in Colorectal Cancer Cells. Cancer Biother. Radiopharm..

[113] Ju J., Schmitz J.C., Song B., Kudo K., Chu E. (2007). Regulation of p53 expression in response to 5-fluorouracil in human cancer RKO cells. Clin. Cancer Res..

[114] Kang H.J., Park H.J. (2009). Novel molecular mechanism for actinomycin D activity as an oncogenic promoter G-quadruplex binder. Biochemistry.

[115] Lu D.F., Wang Y.S., Li C., Wei G.J., Chen R., Dong D.M., Yao M. (2015). Actinomycin D inhibits cell proliferations and promotes apoptosis in osteosarcoma cells. Int. J. Clin. Exp. Med..

[116] Humeau J., Sauvat A., Cerrato G., Xie W., Loos F., Iannantuoni F., Bezu L., Levesque S., Paillet J., Pol J. (2020). Inhibition of transcription by dactinomycin reveals a new characteristic of immunogenic cell stress. EMBO Mol. Med..

[117] Wu H.C., Rerolle D., Berthier C., Hleihel R., Sakamoto T., Quentin S., Benhenda S., Morganti C., Wu C., Conte L. (2021). Actinomycin D Targets NPM1c-Primed Mitochondria to Restore PML-Driven Senescence in AML Therapy. Cancer Discov..

[118] Arango D., Wilson A.J., Shi Q., Corner G.A., Aranes M.J., Nicholas C., Lesser M., Mariadason J.M., Augenlicht L.H. (2004). Molecular mechanisms of action and prediction of response to oxaliplatin in colorectal cancer cells. Br. J. Cancer.

[119] Alcindor T., Beauger N. (2011). Oxaliplatin: A review in the era of molecularly targeted therapy. Curr. Oncol..

[120] Sun F., Cui L., Li T., Chen S., Song J., Li D. (2019). Oxaliplatin induces immunogenic cells death and enhances therapeutic efficacy of checkpoint inhibitor in a model of murine lung carcinoma. J. Recept. Signal Transduct. Res..

[121] Wang X., Ren L., Ye L., Cao J. (2023). Photodynamic therapy augments oxaliplatin-induced immunogenic cell death in colorectal cancer. Cent. Eur. J. Immunol..

[122] Tomicic M.T., Kramer F., Nguyen A., Schwarzenbach C., Christmann M. (2021). Oxaliplatin-Induced Senescence in Colorectal Cancer Cells Depends on p14(ARF)-Mediated Sustained p53 Activation. Cancers.

[123] Ko J.H., Sethi G., Um J.Y., Shanmugam M.K., Arfuso F., Kumar A.P., Bishayee A., Ahn K.S. (2017). The Role of Resveratrol in Cancer Therapy. Int. J. Mol. Sci..

[124] Ren B., Kwah M.X., Liu C., Ma Z., Shanmugam M.K., Ding L., Xiang X., Ho P.C., Wang L., Ong P.S. (2021). Resveratrol for cancer therapy: Challenges and future perspectives. Cancer Lett..

[125] Wang D.J., Liu J.L., Wang J., Feng Y. (2025). Resveratrol protects against uremic serum-induced endothelial cell injury by activating the FUS/KLF2/FBXW7 signaling pathway. Appl. Biol. Chem..

[126] Zhang Y., Yang S., Yang Y., Liu T. (2019). Resveratrol induces immunogenic cell death of human and murine ovarian carcinoma cells. Infect. Agent. Cancer.

[127] Kilic Eren M., Kilincli A., Eren O. (2015). Resveratrol Induced Premature Senescence Is Associated with DNA Damage Mediated SIRT1 and SIRT2 Down-Regulation. PLoS ONE.

[128] Li B., Hou D., Guo H., Zhou H., Zhang S., Xu X., Liu Q., Zhang X., Zou Y., Gong Y. (2017). Resveratrol sequentially induces replication and oxidative stresses to drive p53-CXCR2 mediated cellular senescence in cancer cells. Sci. Rep..

[129] Imran M., Saleem S., Chaudhuri A., Ali J., Baboota S. (2020). Docetaxel: An update on its molecular mechanisms, therapeutic trajectory and nanotechnology in the treatment of breast, lung and prostate cancer. J. Drug Deliv. Sci. Technol..

[130] Pienta K.J. (2001). Preclinical mechanisms of action of docetaxel and docetaxel combinations in prostate cancer. Semin. Oncol..

[131] Flieswasser T., Van Loenhout J., Freire Boullosa L., Van den Eynde A., De Waele J., Van Audenaerde J., Lardon F., Smits E., Pauwels P., Jacobs J. (2020). Clinically Relevant Chemotherapeutics Have the Ability to Induce Immunogenic Cell Death in Non-Small Cell Lung Cancer. Cells.

[132] Zhao S., Xing S., Wang L., Ouyang M., Liu S., Sun L., Yu H. (2023). IL-1beta is involved in docetaxel chemoresistance by regulating the formation of polyploid giant cancer cells in non-small cell lung cancer. Sci. Rep..

[133] Tuli H.S., Rath P., Chauhan A., Parashar G., Parashar N.C., Joshi H., Rani I., Ramniwas S., Aggarwal D., Kumar M. (2023). Wogonin, as a potent anticancer compound: From chemistry to cellular interactions. Exp. Biol. Med..

[134] Polier G., Ding J., Konkimalla B.V., Eick D., Ribeiro N., Kohler R., Giaisi M., Efferth T., Desaubry L., Krammer P.H. (2011). Wogonin and related natural flavones are inhibitors of CDK9 that induce apoptosis in cancer cells by transcriptional suppression of Mcl-1. Cell Death Dis..

[135] Yang Y., Li X.J., Chen Z., Zhu X.X., Wang J., Zhang L.B., Qiang L., Ma Y.J., Li Z.Y., Guo Q.L. (2012). Wogonin induced calreticulin/annexin A1 exposure dictates the immunogenicity of cancer cells in a PERK/AKT dependent manner. PLoS ONE.

[136] Yang D., Guo Q., Liang Y., Zhao Y., Tian X., Ye Y., Tian J., Wu T., Lu N. (2020). Wogonin induces cellular senescence in breast cancer via suppressing TXNRD2 expression. Arch. Toxicol..

[137] Wang Q., Wang J., Wang J., Ju X., Zhang H. (2021). Molecular mechanism of shikonin inhibiting tumor growth and potential application in cancer treatment. Toxicol. Res..

[138] Liang W., Cai A., Chen G., Xi H., Wu X., Cui J., Zhang K., Zhao X., Yu J., Wei B. (2016). Shikonin induces mitochondria-mediated apoptosis and enhances chemotherapeutic sensitivity of gastric cancer through reactive oxygen species. Sci. Rep..

[139] Lee J.H., Han S.H., Kim Y.M., Kim S.H., Yoo E.S., Woo J.S., Jung G.H., Jung S.H., Kim B.S., Jung J.Y. (2021). Shikonin inhibits proliferation of melanoma cells by MAPK pathway-mediated induction of apoptosis. Biosci. Rep..

[140] Chen H.M., Wang P.H., Chen S.S., Wen C.C., Chen Y.H., Yang W.C., Yang N.S. (2012). Shikonin induces immunogenic cell death in tumor cells and enhances dendritic cell-based cancer vaccine. Cancer Immunol. Immunother..

[141] Chen J., Liu J., Liu X., Wang J., Wang X., Ye X., Xie Q., Liang J., Li Y. (2024). Shikonin improves the effectiveness of PD-1 blockade in colorectal cancer by enhancing immunogenicity via Hsp70 upregulation. Mol. Biol. Rep..

[142] Lin T.J., Lin H.T., Chang W.T., Mitapalli S.P., Hsiao P.W., Yin S.Y., Yang N.S. (2015). Shikonin-enhanced cell immunogenicity of tumor vaccine is mediated by the differential effects of DAMP components. Mol. Cancer.

[143] Zheng H., Huang Q., Huang S., Yang X., Zhu T., Wang W., Wang H., He S., Ji L., Wang Y. (2018). Senescence Inducer Shikonin ROS-Dependently Suppressed Lung Cancer Progression. Front. Pharmacol..

[144] Liu W., Zhao Y., Liu Q., Wu D., Li W., Fu Z., Yang L., Liang Y. (2024). Systematic bioinformatics analysis reveals the role of shikonin in blocking colon cancer progression by identifying senescence-induced genes. Front. Pharmacol..

[145] Lyman G.H. (2009). Impact of chemotherapy dose intensity on cancer patient outcomes. J. Natl. Compr. Canc Netw..

[146] Odaimi M., Ajani J. (1987). High-dose chemotherapy. Concepts and strategies. Am. J. Clin. Oncol..

[147] Lim C.-J., Hong J.-Y., Ko Y.-S., Chung M.-W., Jun C.-H., Choi S.-K., Cho S.-B. (2019). High-dose versus Low-dose 5-Fluorouracil and Cisplatin Based Hepatic Arterial Infusion Chemotherapy for Advanced Hepatocellular Carcinoma. J. Liver Cancer.

[148] Selle F., Gligorov J., Soares D.G., Lotz J.P. (2016). High-dose chemotherapy as a strategy to overcome drug resistance in solid tumors. Bull. Cancer.

[149] Ewald J.A., Desotelle J.A., Wilding G., Jarrard D.F. (2010). Therapy-induced senescence in cancer. J. Natl. Cancer Inst..

[150] Majumder P.K., Grisanzio C., O’Connell F., Barry M., Brito J.M., Xu Q., Guney I., Berger R., Herman P., Bikoff R. (2008). A prostatic intraepithelial neoplasia-dependent p27 Kip1 checkpoint induces senescence and inhibits cell proliferation and cancer progression. Cancer Cell.

[151] Choi J., Shendrik I., Peacocke M., Peehl D., Buttyan R., Ikeguchi E.F., Katz A.E., Benson M.C. (2000). Expression of senescence-associated beta-galactosidase in enlarged prostates from men with benign prostatic hyperplasia. Urology.

